# Stochastic effects in adaptive reconstruction of body damage: implied the creativity of natural selection

**DOI:** 10.1111/jcmm.12647

**Published:** 2015-07-08

**Authors:** Bo Xiao, Li-Qiang Cui, Tian-Ming Chen, Bin Lian

**Affiliations:** a Key Laboratory for Ecology and Pollution Control of Coastal Wetlands, School of Environmental Science and Engineering, Yancheng Institute of TechnologyYancheng, China; b Jiangsu Key Laboratory for Microbes and Functional Genomics, Jiangsu Key Lab for Biodiversity and Biotechnology, College of Life Science, Nanjing Normal UniversityNanjing, China

**Keywords:** evolutionary novelty, adaptive evolution, macroevolution and microevolution, stochastic effects, cytoarchitecture

## Abstract

After an injury occurs, mechanical/biochemical loads on muscles influence the composition and structure of recovering muscles; this effect likely occurs in other tissues, cells and biological molecules as well owing to the similarity, interassociation and interaction among biochemical reactions and molecules. The ‘damage and reconstruction’ model provides an explanation for how an ideal cytoarchitecture is created by reducing components not suitable for bearing loads; in this model, adaptive changes are induced by promoting the stochasticity of biochemical reactions. Biochemical and mechanical loads can direct the stochasticity of biochemical reactions, which can in turn induce cellular changes. Thus, mechanical and biochemical loads, under natural selection pressure, modify the direction of cell- and tissue-level changes and guide the formation of new structures and traits, thereby influencing microevolution. In summary, the ‘damage and reconstruction’ model accounts for the role of natural selection in the formation of new organisms, helps explain punctuated equilibrium, and illustrates how macroevolution arises from microevolution.

## Introduction

After physical illness or injury, many patients undergo treatment and hope to recover immediately. They want to regain health and physical functions or even become healthier than they were before the injury occurred. Muscle injury is common in military training, sports training and daily activities. However, recovery often takes a long time, and muscle functions may only be partially restored. Moreover, repetitive injury is highly probable 1. Although self-recovery is the main form of recovery involved in scar healing, skeletal muscle cells do not have regeneration potential 2,3. Nevertheless, proper exercises and favourable biochemical/physical factors can effectively promote the recovery and functional reconstruction of muscles.

Significant efforts have been devoted to prevent and treat skeletal muscle injuries. Studies have reported the use of bioactive molecules, massage and ultrasound to treat such injuries. For instance, insulin-like growth factor-1 is a polypeptide with anabolic and growth-promoting functions; this polypeptide plays a role in the regulation and promotion of muscle recovery 4. Although studies have yet to elucidate whether antioxidants, such as oral vitamins and polyphenols, can help restore muscle functions after an injury, doctors may advise patients to consume antioxidant-containing foods such as blueberries 5. Pulsed ultrasound can promote cell proliferation, protein synthesis and cytokine generation. Thus, recovery speed from skeletal muscle contusion can be accelerated and recovery quality is high 6. Moreover, massage is a common and effective physical rehabilitation method for skeletal muscles. Massage therapy can contribute to muscle recovery by activating cellular mechanotransduction pathways, reducing inflammatory reaction and promoting the biosynthesis of mitochondria 7,8. However, these recovery methods have some limitations, such as toxicity and side effects of chemicals or physiotherapy, unclear mechanism of recovery and disagreement among research results.

Proper post-operative exercises have been clinically demonstrated to promote the recovery of muscles, ligaments and tendons. Researchers have evaluated the increase or reduction in stress load on normal or injured tendons and ligaments. In addition, the effects of exercise on muscle contraction and mitochondrial respiration or on DNA methylation and gene and protein expression have been investigated. Researchers have also focused on the relationship of these molecular changes with tissue reconstruction and functional adjustment 9,10. However, restored muscles may undergo hypertrophy because of overloading induced by surgical removal of congener in the injured skeletal muscle without adjusting stress distribution. Moreover, unloaded activities of the legs and weightlessness in space likely lead to significant loss of muscle mass 11. Adaptive changes in muscles caused by load variation involve a series of physiological responses. Humoral factors, such as growth factors, insulin, adrenal cortex hormones and sex hormones, also affect skeletal muscle mass. Growth hormones can increase bw and muscle mass; by contrast, unloading counteracts the effect of hormones. Under physiological conditions, loading and hormones can regulate muscle mass 12.

Studies conducted to date do not provide a good account of the mechanism by which physical and chemical therapies and exercises affect the repair and functional recovery of muscles after injury. Functional maintenance and injury repair of muscles are jointly affected by several factors. In fact, interactions among such factors play a key role in determining the rate and extent of functional recovery. This is a major reason behind the significant disagreement observed among research results. To optimize post-operative therapy, determining the key points of muscle growth and functional recovery is vital. Hence, establishing basic knowledge on the role of mechanical/biochemical environments in the recovery of attachment points of muscles after injury is paramount.

## Stochasticity of biochemical reactions

During normal cell metabolism, each cell undergoes a large number of biochemical reactions that require a diversity of biological molecules. However, under the constraints of cell space and resources and the need for metabolic orderliness, cells use optimized resources because of low stock and high flux. As a result, although the types of molecules may be highly diverse, only a few copies of each molecule are involved. In addition, the biochemical reactions that occur at the scale of a single cell are influenced by stochastic effects, so they cannot be predicted (because of high intrinsic noise). Furthermore, cell metabolism is affected by dynamic physiological activities, such as fluctuations of the metabolism of transcription factors or hormones. This further increases the extent of uncertainty in biochemical reactions (extrinsic noise) 13,14. The stochastic fluctuation of the physiological microenvironment of cells also directly influences cellular functions, resulting in cellular diversity within the same tissue or organ 15. The stochastic effects of metabolic regulatory networks and the associated biochemical reactions have been shown to promote population diversity. This allows large cell populations to cope efficiently with stress from the external environment and thus confers evolutionary advantages 16,17.

For gene expression, DNA as a template is transcribed into RNA, which is then translated to protein. As such, accurate prediction of protein structure by using DNA sequence data is possible. However, either randomness shown in transcription and translation or dynamic fluctuation of cell components adds to the uncertainty of specific gene expression (noises in gene expression) 18. A single transgenosis event is unlikely to produce the same protein products because of the stochasticity of protein expression and the combination of expression products 19. In turn, stochasticity of gene expression affects biochemical processes. The copy number of many proteins in cells fluctuates randomly, producing perturbations in downstream biochemical reactions. Therefore, even the cells carrying exactly the same genes or cloned under the same environment (genetically identical cells in identical environments) may present phenotypic diversity 20,21. Stochastic effect is observed even in the more precise DNA replication process, which involves the use of restriction enzymes to correct mismatched bases 22.

The stochasticity of biochemical reactions likely becomes more pronounced with ageing. In the middle and late stages of a cell cycle, the disorder of synthesis and allocation of molecules increases. Variation between adjacent cells is also enhanced. For example, the consistency of ageing muscles decreases, and stochasticity strongly influences muscle functions 23,24. The increased stochasticity of DNA replication likely aggravates DNA injury and mutation; thus, the incidence of spontaneous mutation is greater than expected. Pathological problems, such as mitochondrial DNA disorder and other lesions such as tumours or fibrosis, can arise from such mutations. The stochasticity of biochemical reactions may cause cells to become non-viable in spite of the young and energetic body. During ageing, physiological and pathological lesions and changes can accumulate, thereby complicating the stochastic changes in protein expression. For example, diabetes in middle-aged and elderly individuals possibly intensifies non-enzymatic glycosylation already occurring in the human body. Thus, functional decline in proteins facilitates ageing and lesions in organisms 25,26.

Order and disorder of life activities coexist. This study attempts to provide an explanation for the influence of stochastic effects on injury and repair of organisms at a molecular level.

## ‘Damage and reconstruction’ mechanism

Owing to the similarities, interassociation and interaction among biochemical reaction and molecules, the rules of muscle recovery and the stochasticity of biochemical reactions can be extended to other tissues, cells and biological molecules.

**Figure 1:**
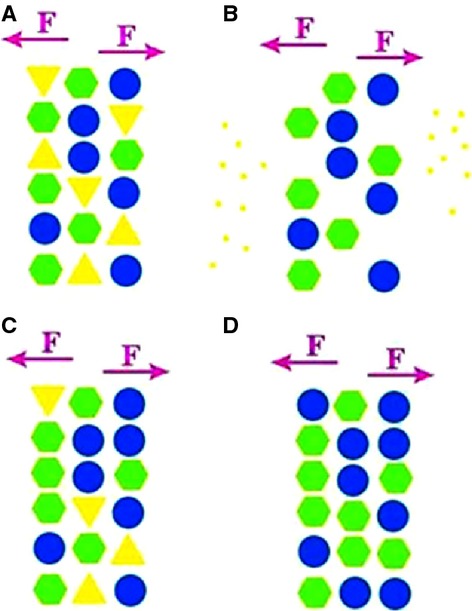
(A) Triangle, hexagon and circle are the assumptive molecular elements of one cytoarchitecture. Triangle represents hard and brittle components with less flexibility; circle represents flexible components; and hexagon represents components with moderate flexibility and brittleness. F represents mechanical load on the cytoarchitecture. (B) Hard and brittle parts represented by triangles are partially damaged, but the basic template of the cytoarchitecture is not affected. (C) After the damaged components are repaired, the ‘triangle’ components decrease because of stochasticity in the repair process; ‘hexagon’ and ‘circle’ components increase. (D) As the random repair process continues, the repaired cytoarchitecture no longer contains hard and brittle parts; in this way, the adaptive repair process is completed.

An ideal cytoarchitecture (or biomacromolecule or muscle tissue; Fig.1) is considered, in which the triangle, hexagon and circle represent the constituent molecules of the cytoarchitecture (compositions of biomacromolecule or molecules with relatively small size). The triangles represent hard and brittle components, which have limited flexibility; circles represent flexible components; hexagons represent components with moderate flexibility and brittleness. This cytoarchitecture bears the action of mechanical or biochemical factors, which can be simplified into a single mechanical load. At a proper level, this mechanical load can damage the constituent components of the cytoarchitecture. In that case, the cytoarchitecture should be repaired, not reconstructed. Considering that the triangles represent hard and brittle components that are partially mechanically damaged, we found that the original framework of cytoarchitecture still exists. An ideal repair can be compared with filling or reproduction by template. After a structure is repaired, the constituent components are completely the same as they were before the injury. However, the cost of complete repair can be hardly bearable in nature under natural selection pressure. The low copy number of molecules is the reason that molecules or precursors necessary for reconstruction are insufficient. The distribution of core molecules required for synthesis provides a framework for the reconstruction of tissues. The stochasticity of molecules with low copy number (in terms of position of molecules and biochemical reactions, such as non-enzymatic glycosylation) results in the variability in microstructures 27. The original hard and brittle constituents of the cytoarchitecture are partially replaced by other constituents during repair because of the stochastic effects of the repair process. As this process continues, the mechanical load on the cytoarchitecture begins to assume the role of guiding the formation of functions or structure. As a result, the cytoarchitecture no longer contains hard and brittle constituents at the completion of adaptive repair.

Another situation is that the load is not specific to one constituent. Instead, the load results in equal damage to all constituents or the cytoarchitecture should be supplemented after consumption related to metabolism. During partial repair or full reconstruction, the cytoarchitecture likely experiences fluctuation of physiological activities, changes in the copy number of remedy molecules, and stochasticity of repair in the template framework. All of these changes likely modify the probability of certain molecules participating in repair-related metabolism or in evolving towards certain repair-related components. The cytoarchitecture is altered after repair, and the generation of new components or structural rearrangement may imply new functions. Environmental changes (environmental load without injury to the cytoarchitecture) cause variation in copy numbers of material molecules of synthesis and repair. Environmental load indirectly influences the structure and functions of the cytoarchitecture, resulting in adaptive changes.

**Figure 2:**
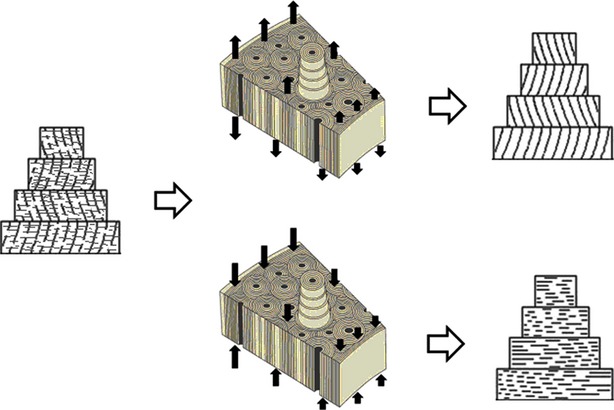
Mechanical loads affect the orientation and arrangement of collagen fibres. Under tension or pressure, the structure and bonds of cytoskeleton and collagen fibre are affected: inadaptable structure and bonds are more easily damaged and replaced by those that adapt to mechanical loads. This figure was adapted from van Oers *et al*. (2015) 31.

From a muscle repair perspective, the posited cytoarchitecture can be the initial composition of muscle tissue after recovery or reconstruction from partial or complete injury. The granulation tissue is formed at the site of injury in a fluctuating physiological environment and *via* stochastic biochemical process. Granulation tissue is formed in a filling mode to sustain the basic physiological integrity of an organism in the early stage of repair. As a form of rapid repair, granulation tissues eventually form scars when these tissues lack guidance of refined functions or specific microstructures. The cytoarchitecture possibly undergoes constant adjustment and adaptive repair under the influence of an external force, such as muscle contraction. Stochastic effects are the driving force behind adjustment of granulation tissues in a certain framework. The repair process ends when the load can no longer damage the constituent molecules and the cytoarchitecture. This process can be seen in Figure2. In granulation tissues or muscle tissues subjected to long-term weightlessness, the initial alignment of collagen fibres is disordered. Then, mechanical loading influences the organization and structure of the cytoskeleton at a cellular level. The inadaptable parts of the cytoskeleton and collagen fibre are selectively damaged and then gradually replaced or changed (‘changed’ means that inadaptable cellular and molecular bonds were replaced by those adapt to mechanical loads), which ultimately direct collagen fibre alignments 28–31. The loading can not only affect muscle fibre arrangements but also directly change structures and compositions of the fibres. The compositions adapted to mechanical loads are kept and become dominant parts, which adaptively change and gradually optimize the mechanical properties of muscle fibres 32,33.

**Figure 3:**
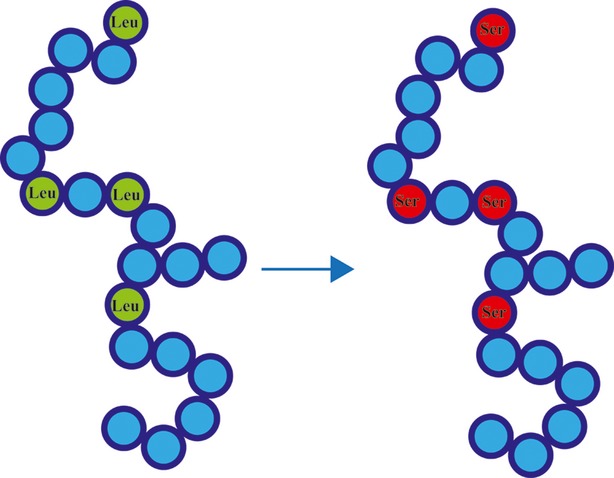
Biochemical loads can modify proteins. When intracellular biochemical environments change, the original protein molecules can be damaged, degraded or metabolized more easily under loads. Environmental changes can also enhance the stochasticity of biochemical reactions and result in mistranslation of the genetic code. Then, in newly synthesized proteins, one amino acid molecule could be occupied by another; for example, Leu could be replaced by Ser, which would be a source of adaptive modification of proteins. This figure was adapted from Moghal *et al*. (2014) 34.

The protein molecules can be modified by biochemical loads generated by changes in intracellular biochemical environments such as oxidation of cellular amino acid pools. The changes in biochemical environments also enhance the stochasticity of biochemical reactions and reduce the accuracy of mRNA translation, resulting in mistranslation of the genetic code. The proteins can be selectively damaged and degraded by loads or metabolized. Then, in newly synthesized proteins, the sites of one amino acid could be occupied by another amino acid, such as replacement of Leu by Ser, which might lead to adaptive changes in the structure and function of proteins (Fig.3) 34–36.

**Figure 4:**
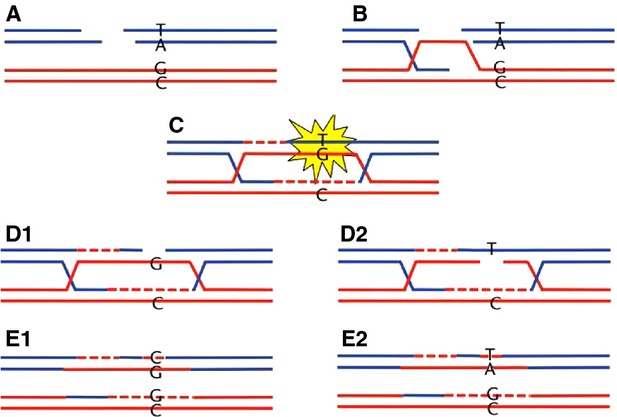
Formation of CG-bias induced by biochemical loads. During meioses, a double-strand break might be initiated on one of two sister chromatids containing more AT (A). The broken sister strand would then invade the intact strand (B). A Holiday junction would be formed and the mismatches would trigger a base-excision repair system (C). During mismatch repair processes, the mismatch would have half the chance of resulting in substitution from A/T to G/C (D and E). This figure was adapted from Backström (2014) 38.

In both mammals and birds, there is GC bias, which can be interpreted as GC-biased mismatch repairing trend (using G or C as the template and cut-off A/T) in the mismatch repair process. It is difficult to explain how the biochemical loads directly and accurately act on mismatched bases, always eliminating one base and preserving another 37–39. Biochemical loads can act on double-stranded DNA and make it easy to break 40. Compared with a single pair of mismatched bases, biochemical loads can affect a section of double-stranded DNA (many pairs of bases) more easily. If the process ‘GC-biased gene conversion’ could be driven by biochemical loads in cellular microenvironments, the breaks would be more likely to occur within one nucleic acid chain with high AT composition under loads (Fig.4). Thus, in the process of mismatch repair, two possible results without bias can occur (Fig.4D and E). There is 50% chance of the mismatch resulting in substitution from A/T to G/C. Then, the GC bias in the reconstruction regions could be induced by selective breaking of AT enrichment regions in nucleic acid chains under biochemical loads. If the biochemical loads brought selective pressures on GC-enriched regions and made them more likely to break, this would explain the AT bias found in mitochondrial genomes 41.

Randomness causes uncertainty in the development and repair at single cell level because microenvironments for cellular biochemical reaction cannot be the same. At a living organism level, the mechanical loading or environmental factors can be observed almost at the same level; all of the repaired parts are also similar to unrepaired ones, including components and tissue structure. This is possible because a closely coordinated development and repair mechanism is established in the chaotic interaction of biomolecules amid the contradiction of microrandomness and macrodecisiveness 42. Changes in the macroenvironment and individual ways of life likely cause rapid alterations in the physiological factors in the internal environment (biochemical load). Such changes may further affect development, growth or repair, which transform the form, structure and function of tissues and organs; as a result, this process drives the adaptive evolution of species.

## Biochemical templates and guiding force

In a physical model, stress concentration possibly forms on the interface to cause fracture when the load is transmitted between relatively soft, elastic tissues and relatively hard, inelastic tissues because these two types of tissues exhibit different mechanical characteristics. A tendon-bone interface is an example of a site with mechanical weakness. Under physiological conditions, a four-layer structure with no clear boundaries exists on the tendon-bone interface. The gradual transition of mechanical properties effectively avoids stress concentration, providing the tissues in this area immense tensile strength and ensuring its load bearing and athletic ability. The tendon-bone interface is reconstructed to recover normal composite structure. Researchers have not been able to guarantee that tendons and bones can bond solidly or avulsion can be avoided completely because the tendon-bone interface structure is complex and the effect of stochasticity on adjustive repair is limited in the range of granulation tissue 43. If similar four-layer templates exist in the early filling repair process, then the load in the adaptive repair process can effectively optimize the structure of new tissues and eliminate the parts with low tensile strength in cytoarchitectures. Thus, tendon reconstruction can be gradually optimized 44.

Differences in the directive force of collagens or several biochemical changes before linking (mechanical/biochemical loads) likely result in the observed differences in the function of collagen fibres. The recombination of collagen fibre possibly enables adaptive changes in tendons under further action of loads. Components, templates and environment for ‘damage and reconstruction’ also likely change as the tendon ages or after the matrix proteins are glycosylated; likewise, the rearrangement rules of collagen fibres may change over time 45–47.

For sedentary white-collar workers, the reduction in loads on the skeletal muscle of legs significantly decreases protein synthesis related to contraction 48. The weightless environment in outer space can cause amyotrophy and partial dysfunction in the soleus and the gastrocnemius muscles of the calf. Sufficient treadmill running can help maintain a few functional enzymes in the muscle cells but cannot avoid the reduction in the expression of several other proteins; thus, weightlessness-caused fatigue arises 49–51. As weight acts on each cell, motion cannot simulate gravity load. Under weightless circumstances, the damage/consumption caused by the action of gravity is reduced; molecular repair/supplement and metabolic rate of relevant proteins in the cytoarchitectures are also decreased. The updated molecules for tissue metabolism cannot be effectively connected and utilized because of the lack of guidance from gravity, resulting in excessive copy number, reduced demand of relevant proteins and further decrease in synthetic rate. The decreased proportion of these proteins in muscle fibres is manifested as muscle mass loss 52.

Therefore, an ideal therapy requires the reconstruction of efficient biochemical templates and the guidance of loads. Post-operative exercise is therefore beneficial for the repair of injured muscles. However, the guiding force in a general exercise regimen is very rough with only a few points of action. More subtle and comprehensive guiding force and efficient biochemical templates may be obtained with the development of medical devices and consumables.

## Physiological environment, diet and exercise

The lack of biological molecules, such as biglycan and decorin, may directly influence the repair of an injured tendon 53. The lack of cellular elements causes limitations in repair materials; thus, the stochasticity (referring to ‘not completely conforming to the templates’) of the repair process increases, and the components of the cytoarchitectures are easily substituted in this process. However, if the copy number of certain cellular element increases, then the stochasticity of repair likely increases the probability of a molecule participating in repair metabolism or becoming a repair component. Cytoarchitectures possibly undergo substitutional changes after repair. Changes in surroundings and individual survival mode likely trigger alterations in the microscopic physiological environment, structural composition and metabolic cellular processes. These changes are manifested as adaptive changes in cells. The environment not only selects but also guides the changes in cell traits. The difference in the order of magnitude of amino acids or the unique precursor of anabolism likely causes differences in the number of corresponding amino acids in a cell protein; thus, nutrients directly affect the physiological environment of cells 54. Reducing the intake of methionine can alter the composition of tight junctional claudin and reduce the conversion of sugar to fat. This process can prolong the lifespan of rodents; notably, this process can also be achieved by dieting 55,56. With the same fat intake, a diet containing high amounts of saturated fat is more likely to cause obesity; by contrast, a vegetarian diet can facilitate weight loss among individuals because of high unsaturated fatty acid content and incomplete essential amino acid profile 57,58.

Mechanical load and internal physiological environment (biochemical load) affect the process and outcome of cellular metabolism and ‘damage and reconstruction’. Fat accumulates in body parts with less muscular activity. Proper exercise can enhance the contractility of exercising muscles. Fat cannot be stored in active muscle cells after the ‘damage and reconstruction’ process because of the ‘loads’ of contraction and acute respiration. Therefore, fat is distributed unevenly over the whole body 59–61. The current authors also hold that the insulation function of subcutaneous fat is favourable for the survival of cold-resistant animals, and the thick fat deposit in cold-resistant animals is the outcome of natural selection in cold high-altitude regions. However, this result is not selected by ‘smart’ genes but is instead caused by the stochastic effects of the ‘damage and reconstruction’ process before genomic change occurs. Melittin is often used to treat diseases such as multiple sclerosis. Melittin can change the internal environment of the human body to produce biochemical loads that affect such diseases. However, the precondition for its application is that its toxicity to the human body should be a secondary, not primary, load 62,63.

Regular exercise can reduce the amount of proteins under oxidative modification in the brain to maintain and improve cognitive functions and to mitigate oxidative stress over the entire body. Diet and exercise can enable functional tissues to form and maintain functional adaptation under the effect of ‘damage and reconstruction’, which promotes good health 64. The active components of natural medicines, such as traditional Chinese medicines, can also enhance endogenous healing of muscle tendons by regulating physiological conditions. Natural medicines combined with early active or passive functional exercises can prevent the stiffness of healing tissues, reduce tendon adhesion and improve local blood circulation to promote muscle function recovery 65.

## Stochastic and active evolution

Although the ‘Damage and Reconstruction’ mechanism and the stochasticity of biochemical reactions are reasonable in physiology and seemingly unrelated to evolution and hereditability, the genetic system and inherited molecules could be influenced by external loads and random biochemical reactions, instead of being standalone and impervious, owing to the similarity, interassociation and interaction among biochemical reactions and molecules.

Currently, evolutionary mechanisms such as transformation and diversification remain poorly understood 66. For example, an effective combination should be established between the mechanisms of macroevolutionary and microevolutionary processes. The relationship between natural selection and change in biological adaptation should be elaborated, particularly the mechanism by which new structures and organs are formed 67–69.

Evidence has shown the significant role of natural selection at molecular evolution levels. Molecular mutations are manifested as either ‘favourable mutations’ or ‘unfavourable mutations’ under different environmental conditions. During the critical stages of species formation, DNA molecules are under positive selection pressure, resulting in sharp increase in the replacement rate of basic groups and corresponding genomic adjustment. Thus, organisms generate a series of microevolutions to affect macroevolution 70,71. A reasonable evolutionary theory should be applied to the chemical diversity of organisms because biodiversity should be supported by biochemical diversity at a molecular level. However, the mechanism of natural selection at a molecular level remains unclear; moreover, the transition from microevolution to macroevolution is imperfect because biologists from the biochemical field are the ones mainly developing evolutionary theory 72.

Natural selection can eliminate unsuitable traits, and individuals in numerous species evolved from a common ancestor. Current evolutionary theories indicate that individual morphological diversity caused by development factors is not associated with natural selection. This concept complicates the knowledge regarding the role of natural selection in evolutionary changes, especially in the evolution of complex tissues, new functions or traits. Therefore, current situations, such as the disapproval of modern synthesis on developmental biology and ecology, impede the integration of evolutionary developmental biology (evo-devo) and evolutionary ecology into the existing theoretical system of evolutionary biology 73–75. If the evolutionary direction is random, then natural selection can only eliminate unsuitable species. With an older population, species diversity becomes high. The evolution of individuals is not random and the differentiation rate of species is affected by the lifestyle of a given population and its surrounding environment. Therefore, the formation time of population cannot determine species diversity, and no evidence has been provided to support the claim that old groups have more species than young groups 76,77. Under any circumstance, a new biological structure evolves according to the hereditary basis provided by the genetic regulation loop of early multicellular animals. Different living modes or environmental changes may cause developmental biases of organisms to form marked phenotypic differences and create the possibility of new structures and new traits 67,78,79. The gradual accumulation of numerous long-term changes is the root of the beginning and evolution of new things, such as the emergence of new cell types, adaptive functional changes and the formation of organs and limbs of multicellular animals. New problems emerge if natural genetic engineering is non-random and sensitive to external input; such problems also arise if natural genetic engineering provides all molecular tools for genomic regulation, that is, this technology is the real source of complicated evolution. The question that should be answered then is, ‘How does microevolution coordinate with macroevolution and how does natural selection play a role in this process?’ 80.

Based on Darwin's theory of natural selection, long-necked giraffes are reproduced because their short-necked ancestors were constantly eliminated. However, further studies should be conducted to explain that natural selection not only eliminates functions but also creates favourable traits. Environmental changes produce the force of natural selection and cause the changes in the individual way of life. These changes possibly affect the biochemical, metabolic and mechanical environment of individuals, thereby inducing selection force to transition into loads. This study argues that an organ or a tissue is likely under pressure from biochemical/mechanical loads in its functioning process, as well as the processes of normal replacement of physiological metabolism and repair replacement after injury. Biochemical reactions include randomness in the process of reproduction of biomolecules; thus, the loads can act on the cytoarchitectures of cells to trigger relevant changes. The stochastic aspect of biochemical reactions creates the possibility of changes in cellular elements; by contrast, mechanical and biochemical loads under the influence of natural selection provide the direction for such changes. We state this inference based on the influence of survival mode. During development, the ancestors of giraffes stretched out their necks and relevant tissues, such as bones, spatium interosseum and muscles, inevitably damaging their less-elastic body parts. In the repair process, filling repair forced the necks to elongate slightly. The random effect of ‘damage and reconstruction’ may increase the components contributing to the elasticity of necks as the factors limiting the stretching of necks are reduced. Under the influence of such process, biochemical reactions in cells, such as DNA duplication, transcription and expression, changed gradually; this change was ultimately manifested as the formation of a new species. The organs/tissues undergoing ‘damage and reconstruction’ possibly utilize more limited resources in the body, reducing the resources necessary to maintain other tissues. This process may be regarded as the use and disuse theory of an individual and at a molecular level may be under the influence of population-level natural selection. The development process and its biochemical reaction likely bear and adapt to selective pressure to a certain extent when the development direction is affected by changes in the environment and survival modes. Individuals and cellular elements possibly exhibit adaptive changes to environmental loads. Such adaptive changes in biochemistry and genetic molecules tend to further affect the subsequent generation, and these changes are manifested as genetic variation. Mature individuals still maintain the ‘damage and reconstruction’ process except the developmental stage of rapid growth but at a slower pace. However, this process is insufficient to explain the mechanism by which the ecological environment and corresponding survival modes affect development and evolution. Nevertheless, our explanation is simple, clear and rational.

We also consider the following assumption. In the age of mass extinction, many species were eliminated because of their inability to adapt to extreme environmental changes. Tremendous changes in both the internal and external environments of living organisms and reconstruction of food chains caused changes in the survival modes of the surviving species. Biochemical changes in cells suffered from greater loads. Environmental changes altered the composition of raw materials involved in biochemical reactions, which likely limited the resources and energy necessary to maintain the original biochemical reaction and increased the disorder of synthesis and distribution of biochemical molecules (similar to the case in which ageing increases uncertainties in biochemical reactions). As stochasticity in ‘damage and reconstruction’ increased, existing cytoarchitectures were altered and more adaptive changes possibly appeared in living organisms at a faster rate. Consequently, species explosion likely occurred after mass extinction. It has been proposed that biological evolution can suddenly accelerate after drastic environmental changes, effectively promoting biological divergence 81,82. Gradual evolution in normal environments and saltational evolution after drastic environmental changes are both necessary to explain the complexity and diversity of life on Earth 83. This explanation of a ‘damage and reconstruction’ mechanism for punctuational evolution is of great theoretical significance in understanding microevolution, its ecological process, and macroevolution 70.

## Conclusion

The stochastic effect of ‘damage and reconstruction’ can provide a theoretical basis to treat diseases and improve post-operative recovery of body damage. It can also provide important inferences for the development of new medicine. Although evolution is not evident in the lifetime of an individual, we can adjust our physical health by engaging in proper diet and exercise. For normal physiological conditions without significant changes in the living environment or diet, the stochastic effect of the ‘damage and reconstruction’ process gradually reduces the components damaged by loads, forms adaptive repair of cytoarchitectures, and facilitates adaptive recovery of muscle functions. Once the environment and survival mode change, mechanical/biochemical loads are possibly generated under the action of natural selection. Under the guidance of such loads, the stochasticity of repair likely promotes the replacement of former cellular elements in a physiological environment and induces compositional and functional changes in the repaired cytoarchitectures. Thus, microevolution occurs and may lead to macroevolution. Furthermore, remarkable changes in living environments and rapid increase in random factors implicated in tissue repair can cause abrupt acceleration of evolution. Thus, this model provides support to the punctuated equilibrium theory at a molecular level. Natural selection not only eliminates unsuitable traits but also guides the formation of new and favourable characteristics. Therefore, the proposed ‘damage and reconstruction’ mechanism offers a rational explanation for the organic combination of microevolution and macroevolution.
